# Identification of blood immunological biomarkers of SARS-CoV-2 infection during pandemic in Poland

**DOI:** 10.3389/fimmu.2025.1613629

**Published:** 2025-09-22

**Authors:** Monika Leśniak, Agata Borkowska, Krzysztof Kłos, Karolina Aleksandrowicz, Klaudia Porębska, Dagmara Kobza, Krzysztof Łukasz Piwowarek, Katarzyna Plewka-Barcik, Marcin Niemcewicz, Anna Lutyńska, Jacek Z. Kubiak, Andrzej Chciałowski, Robert Zdanowski

**Affiliations:** ^1^ Laboratory of Molecular Oncology and Innovative Therapies, Military Institute of Medicine-National Research Institute, Warsaw, Poland; ^2^ Department of Internal Medicine, Infectious Diseases and Allergology, Military Institute of Medicine- National Research Institute, Warsaw, Poland; ^3^ BioMedChem Doctoral School of the University of Lodz and Lodz Institutes of the Polish Academy of Sciences, University of Lodz, Lodz, Poland; ^4^ Biohazard Prevention Centre, Faculty of Biology and Environmental Protection, University of Lodz, Lodz, Poland; ^5^ Dynamics and Mechanics of Epithelia Group, Institute of Genetics and Development of Rennes (IGDR), Faculty of Medicine, University of Rennes, Centre National de la Recherche Scientifique (CNRS), Unité Mixte de Recherche (UMR) 6290, Rennes, France

**Keywords:** SARS-CoV-2, CXCL10, T lymphocytes, COVID-19, severity, biomarkers, pandemic

## Abstract

**Intoduction:**

T lymphocytes, along with cytokines and chemokines-dependent pathways are primarily responsible for regulating the immune response, controlling inflammation and eliminating viral infections. However, excessive immune activity can lead to pathological effects such as cytokine storm, which may cause severe respiratory distress syndrome and multi-organ damage in COVID-19. The aim of this study was to identify potential biomarkers of SARS-CoV-2 infection that could predict the severity of COVID-19 progression.

**Methods:**

The cohort in this study included 52 hospitalized adult patients with SARS-CoV-2 infection from Warsaw, Poland admitted to the hospital during COVID-19 pandemic (February to November 2021). Based on clinical symptoms, patients were divided into two groups: (i) mild/moderate symptoms (non-severe) – 44 patients and (ii) severe respiratory failure (severe) – 8 patients. The control group consisted of 26 individuals without COVID-19. All COVID-19 patients and healthy controls underwent immunophenotyping of peripheral blood to assess the abundance of T lymphocytes and regulatory T lymphocytes, as well as measurement of selected cytokine and chemokine concentrations in corresponding serum samples. Data analysis was performed using CytoFLEX Flow Cytometer.

**Results and discussion:**

Decreased percentages of total lymphocytes and T lymphocytes in peripheral blood were observed across all COVID-19 patients, with varying degrees between the non-severe and severe groups. A significant reduction was also noted in double-positive lymphocytes (CD4+CD8+), regulatory T lymphocytes ( CD4+ CD25HiCD127Lo and CD4+CD25HiCD127LoFoxP3+), as well as CD4+CD25+/-, CD4+CD45RA+/-, and CD8+CD45RA+/- subsets. Elevated levels of IL-6, IL-10, IL-17A, IFN-g, CCL2, CXCL8, and CXCL10 were observed in the non-severe and/or severe groups compared to healthy controls. Most importantly, only CXCL10 was significantly elevated in the severe group at admission compared to the non-severe group. In this study, we identified the chemokine CXCL10 as a crucial marker for distinguishing the severe course of COVID-19 from non-severe form at the time of admission. It may serve as an early indicator of diseases progression during hospitalization, potentially allowing prediction of the disease course. Moreover, elevated CXCL10 levels, in combination with decreased total lymphocytes counts and increased levels of IL-6, IL10, IFNg, CCL2 and CXCL9, may represent a more comprehensive biomarker panel suitable for predicting the severity of COVID-19.

## 1 Introduction

SARS-CoV-2 is a highly transmissible and pathogenic coronavirus, emerged at the end of 2019, causing the global COVID-19 pandemic. The disease presents with a wide spectrum of symptoms, ranging from mild flu-like symptoms to severe, life-threatening conditions including cytokine storms. Initial clinical symptoms such as: fever, general weakness, and/or muscle pain (1), are often accompanied by shortness of breath, fatigue, and/or loss of taste and smell (2). Direct contact, including transmission through secretions and droplets from the nasal and oral mucosa during coughing or sneezing is widely recognized as a primary route of infection (3).

Coronaviruses are pleomorphic particles with a spherical envelope composed of a two-layered lipid structure embedded with structural membrane (M), envelope (E), and spike (S) proteins, and containing a positive-sense RNA genome. The spike glycoprotein, composed of subunits S1 and S2, includes a receptor-binding domain (RBD) responsible for interacting with the host cell receptor (1, 4). The SARS-CoV-2 spike glycoprotein binds the angiotensin-converting enzyme 2 (ACE-2), a membrane bound carboxypeptidase expressed in various human tissues (5). Following viral entry, the innate immune response acts as the first line of defense, employing multiple strategies to detect and eliminate the virus. Natural Killer cells (NK), macrophages, monocytes, dendritic cells, and neutrophils are rapidly activated and play a critical role in generating inflammatory cytokines, such as type I interferons (6).

SARS-CoV-2 primarily replicates in the epithelium of the upper respiratory tract, leading to the activation of antigen-presenting cells (APCs), and subsequently, the activation of naïve CD4^+^ and CD8+ T as well as memory T cells. Activated effector T lymphocytes migrate to the infection sites and play a crucial role in the antiviral immune response. CD4^+^ T cells promote macrophage activation, stimulate antibody production by B cells and assist CD8+ T cells in developing effective cytotoxicity response (7). CD8^+^ T cells, upon recognition of viral peptides via presented via Major Histocompatibility Complex Class I (MHC I), contribute to the formation of SARS-CoV-2-specific memory T cells and induce apoptosis in infected cells through the activation of perforin and granzymes. Extensive activation of macrophages, monocytes, neutrophils, and T lymphocytes, along with elevated levels of cytokines and chemokines, such as IFNα, IFNγ, IL-1β, IL-6, IL-12, IL-18, IL-33, TNFα, TGFβ, CCL2, CCL3, CCL5, CXCL8, CXCL9, and/or CXCL10 leads to a strong inflammatory response, which can culminate in a cytokine storm, acute respiratory distress syndrome (ARDS) and multiorgan failure (8). B lymphocyte independently recognize viral antigens and subsequently proliferate and differentiate into antibody-producing plasma cells and memory B cells (9).

Although knowledge and experience related to COVID-19 grown exponentially in recent years, a deeper understanding of the immunopathology associated with the SARS-CoV-2 infection remains desirable and is recognized as a global health challenge (10). Effective management and treatment of SARS-Cov2 still require improvement, particularly in predicting potentially severe conditions in infected patients at the time of admission. A significant decrease in overall lymphocyte count, especially T cells has been widely observed in the blood of COVID-19 patients (11). In severe cases, a marked deficiency of eosinophils and lymphocytes, significantly reducing the number of T, B, and NK cells, has also been reported (12). This profile is specific to the blood of affected patients and is, at least in part, related to the migration of these immune cells to the lungs (13).

Our study examines changes in T lymphocytes, specific T lymphocytes subsets, including: CD4^+/-^CD8^+/-^, CD4^+^CD25^+/-^, CD8^+^CD25^+/-^, CD4^+^CD45RA^+/-^, CD8^+^CD45RA^+/-^, CD4^+^CD25^Hi^CD127^Lo^, CD4^+^CD25^Hi^CD127^Lo^FoxP3^+^, as well as selected cytokines/chemokines in COVID-19 patients and healthy controls. In this study, samples were collected from COVID-19 patients hospitalized at the Military Institute of Medicine – National Research Institute (MIM-NRI) in Warsaw between February and May 2021. Samples from healthy individuals recruited between February and November 2021 were also used. The aim of the study was to identify a panel of potential biomarkers associated with COVID-19 severity in order to enhance the understanding of its immunopathology and to propose future diagnostics tools capable of predicting the severity of the disease course.

## 2 Materials and methods

### 2.1 Patient cohort

This prospective observational cohort study involved hospitalized COVID-19 patients, with data and sample collection initiated at the time of hospital admission to the Department of Internal Medicine, Infectious Diseases, and Allergology of the Military Institute of Medicine – National Research Institute in Warsaw, Poland. The inclusion criteria were: the age over 18 years, a positive SARS-CoV-2 real-time PCR test at the time of admission, availability of initial chest CT (computed tomography) scans showing typical COVID-19 related lung lesions, and the ability and willingness to provide informed consent to participate in the study. The study was approved by the MIM-NRI Ethics Committee (approval No: 3/WIM/2021, dated 20 February 2021), and all enrolled patients provided written informed consent.

We examined a cohort of 52 unvaccinated, hospitalized adult patients with COVID-19. For baseline comparison, a control group of 26 individuals (21 vaccinated and 5 unvaccinated) was included., All controls were PCR-negative for SARS-CoV-2 and confirmed negative for antibodies using Polycheck Anti-SARS-CoV-2 IgG Immunoblot (Polycheck, Germany). Patients were divided into two groups according to severity grades described by the World Health Organization (WHO) (14). Disease severity was assessed retrospectively based on disease progression. The non-severe course COVID-19 group consisted of 44 patients with mild or moderate disease symptoms, clinical signs of pneumonia and peripheral oxygen saturation (SpO2) >90%. The severe course COVID-19 group consisted of 8 patients with severe disease symptoms, characterized by pneumonia and SpO2 < 90%, respiratory rates > 30 breaths/min, or critical disease, including progression of pulmonary imaging lesions > 50% within 24–48 h, ARDS, sepsis, or septic shock. Importantly, five patients in the severe group died during hospitalization. Demographic and clinical characteristics of the examined groups is presented in **Table 1**.

**Table 1:** Demographic and clinical characteristics, comorbidities, hospitalization and treatment of the study cohort.

Characteristic	Healthy patient (n = 26)	Disease severity (WHO ordinary scale)	
		Patients non-severe (WHO 4 – 5; n = 44)	Patients severe (WHO 6 – 7; n = 8)
Age, median ± SD - yr	53,96 ± 13,45	56,86 ± 12,38	65,75 ± 12,61
Sex – n (%)			
Male - n (%)	12 (46)	23 (52)	4 (50)
Female – n (%)	14 (54)	21 (48)	4 (50)
Body-mass index - median (SD)	30,7 ± 6,94	31,5 ± 6,63	30,4 ± 6,12
Obesity – n (%)	15 (58)	27 (61)	5 (62)
Tabaco use – n (%)	8 (31)	13 (30)	1 (13)
Comorbidities			
All comorbidities – n (%)	10 (39)	29 (66)	7 (88)
Asthma – n (%)	0	4 (9)	1 (13)
COPD – n (%)	0	1 (2)	0
Chronic kidney disease – n (%)	0	1 (2)	0
Hypertension – n (%)	10 (39)	15 (34)	4 (50)
Coronary artery disease – n (%)	0	4 (9)	1 (13)
Heart failure – n (%)	0	1 (2)	1 (13)
Atrial fibrillation – n (%)	1 (4)	3 (7)	0
Cancer – n (%)	0	3 (7)	0
Diabetes type 2 – n (%)	0	6 (14)	4 (50)
Hospitalization			
Time from onset of COVID-19 symptoms to hospital admission – days (± SD)	NA	6,7 ± 3,4	9,5 ± 5,53
Time to hospital discharge – days (± SD)	NA	14,7 ± 7,42	18 ± 9,17
Days until progression to severe disease – days (± SD)	NA	NA	2,4 ± 1,41
Treatment of COVID-19 and complications			
Remdesivir – n (%)	NA	27 (61)	4 (50)
Dexamethasone – n (%)	NA	34 (77)	8 (100)
Low molecular weight heparin – n (%)	NA	42 (96)	8 (100)
New oral anticoagulants – n (%)	1 (4)	2 (5)	0
Antibiotics (ceftriaxone, levofloxacin, piperacillin/tazobactam) – n (%)	NA	35 (80)	8 (100)
Budesonide (inhalation) – n (%)	NA	8 (18)	2 (25)

Samples from COVID-19 patients were collected within 24 hours of hospital admission, at a time, when there was no indication that the patient was in a severe or life-threatening condition. Samples from healthy individuals were collected from volunteers, who did not show symptoms of infection and had not been hospitalized at the time of the study. Flow cytometry analysis of T lymphocyte populations was carried out on samples collected from 44 COVID-19 patients (38 non-severe group, 6 severe group) and 25 healthy individuals. Flow cytometry analysis of regulatory T lymphocyte populations was performed on samples collected from 39 patients (34 in the non-severe group, 5 in the severe group) and 25 healthy individuals. Cytokine and chemokine concentrations were analyzed in samples collected from 52 patients (44 in the non-severe group, 8 in the severe group) and 26 healthy individuals. The differences in group size are due to lack or insufficient quality of the collected biological material.

### 2.2 Immunophenotyping of peripheral blood cells

Whole venous blood samples were collected into EDTA tubes. The antibodies used for T lymphocyte and regulatory T lymphocyte panels are presented in **Table 2**. Antibodies and 100 µl of whole blood samples were added to cytometric tubes (Falcon, USA) and incubated for 30 minutes in the dark at room temperature (RT). After labeling erythrocytes were lysed with 1 ml FACS Lysing Solution (BD Bioscience, USA) and incubated for 10 minutes in the dark in RT. Subsequently, the surviving cells were washed with PBS solution (Corning, USA). Next, the cells were fixed with 4% paraformaldehyde (PFA) (Merck, Germany) for 5 minutes in the dark at RT and washed with PBS. Intracellular staining for FoxP3 was performed using the PerFix-NC Kit (B31168, Beckman Coulter, USA) according to manufacturer’s protocol. After labeling, samples were re-suspended in 200 µl of PBS and analyzed in 24 hours using a CytoFLEX Flow Cytometer (Beckman Coulter, USA,2019) and CytExpert version 2.3.0.84 software (Beckman Coulter, USA). The samples were stored in the darkness at 4°C until analysis.

**Table 2:** Antibodies used in the flow cytometric panels in the study.

Populations	Product description	Cat. no.	Manufacture	Clone
T lymphocytes panel	CD3-APC-AF750	A94680	Beckman Coulter, USA	UCHT1
	CD4-APC	IM2468U	Beckman Coulter, USA	13B8.2
	CD8-APC-A700	B49181	Beckman Coulter, USA	B9.11
	BV785 Mouse Anti-Human CD25	563700	BD Horizon, USA	M-A251
	CD45-Krome Orange	B36294	Beckman Coulter, USA	J33
	CD45RA-FITC	A07786	Beckman Coulter, USA	ALB11
regulatory T lymphocytes panel	CD3-APC-AF750	A94680	Beckman Coulter, USA	UCHT1
	CD4-APC	IM2468	Beckman Coulter, USA	13B8.2
	CD45-Krome Orange	B36294	Beckman Coulter, USA	J33
	BV605 Brilliant Violet 605 anti-human CD19 Antibody	302244	BioLegend, USA	HIB19
	Brilliant Violet 785 anti-human CD127 (IL-7Rα) Antibody	351330	BioLegend, USA	A019D5
	R718 Mouse Anti-Human FoxP3	566937	BD Bioscience, USA	259D/C7

### 2.3 Sera collection

Whole blood was collected into tubes with a separation medium and centrifugated at 2000×g for 20 minutes at RT. The serum was separated, aliquoted into sterile 1.5 mL tubes (Eppendorf, USA), and stored at -80°C for future analysis.

### 2.4 Cytokines/chemokines profile

Cytokine and chemokine profiles were determined using the BD Cytometric Bead Array (CBA) Human Th1/Th2/Th17 Cytokine Kit (IL2, IL4, IL6,IL10, TNF, IFNγ, IL17A; ref. 560484) and Human Chemokine Kit (CCL2/MCP1, CCL5/RANTES, CXCL8/IL8, CXCL9/MIG, CXCL10/IP-10; ref. 552990), both from BD Bioscience, USA. Undiluted serum was used for the analysis. A standard curve consisting of 10 points, with concentrations ranging from 0 to 5000 pg/mL (0, 20, 40, 80, 156, 312.5, 625, 1250, 2500, 5000) for cytokines and 0 to 2500 pg/mL (0, 10, 20, 40, 80, 156, 312.5, 625, 1250, 2500) for chemokines was prepared.

Capture beads were vortexed thoroughly and combined into a single solution. For cytokines determination, the capture beads were centrifuged (200 RCF, 5 minutes, RT), and the supernatant was discarded. The pellet was resuspended in a Serum Enhancement Buffer and incubated for 30 minutes at RT in the dark. This step was omitted in analyzes for chemokine determination.

Next, capture beads, serum, and detection reagent were added to cytometric tubes and incubated for 3 hours at RT in the dark. The samples were then washed and resuspended in a wash buffer. The prepared samples were immediately analyzed using a Cyto FLEX Flow Cytometer (Beckman Coulter, USA) and processed with FCAP Array Software Version 3.0.1 (BD Biosciences, USA).

### 2.5 Statistical analysis

GraphPad Prism software (v. 10.4.1, La Jolla, CA, USA) was used for statistical analysis. The normality of distributions was assessed using the Shapiro–Wilk test. Depending on the results, comparisons between groups were performed using either the unpaired t-test (with Welch’s correction when variances were unequal) or the Mann–Whitney U test for non-normally distributed data. For comparisons involving three groups, appropriate pairwise tests were applied. Since multiple comparisons were performed (cytokines, lymphocyte subpopulations), p-values were adjusted for multiple testing using the Benjamini–Hochberg procedure to control the False Discovery Rate (FDR). Only FDR-adjusted p-values are reported, and statistical significance was defined as FDR-adjusted p < 0.05.

### 2.6 Correlation matrix

Variables that showed statistical significance in the study were selected for correlation analysis. A correlation matrix was prepared using GraphPad Prism (v. 10.4.1, La Jolla, CA, USA). Spearman’s rank correlation (Spearman’s R test) was used to analyze correlations for nonparametric distribution. A p-value of ≤0.05 was considered statistically significant.

## 3 Results

### 3.1 Total lymphocytes and double-positive CD4^+^CD8^+^ lymphocytes populations decrease in COVID-19 patients

**Figure 1:**
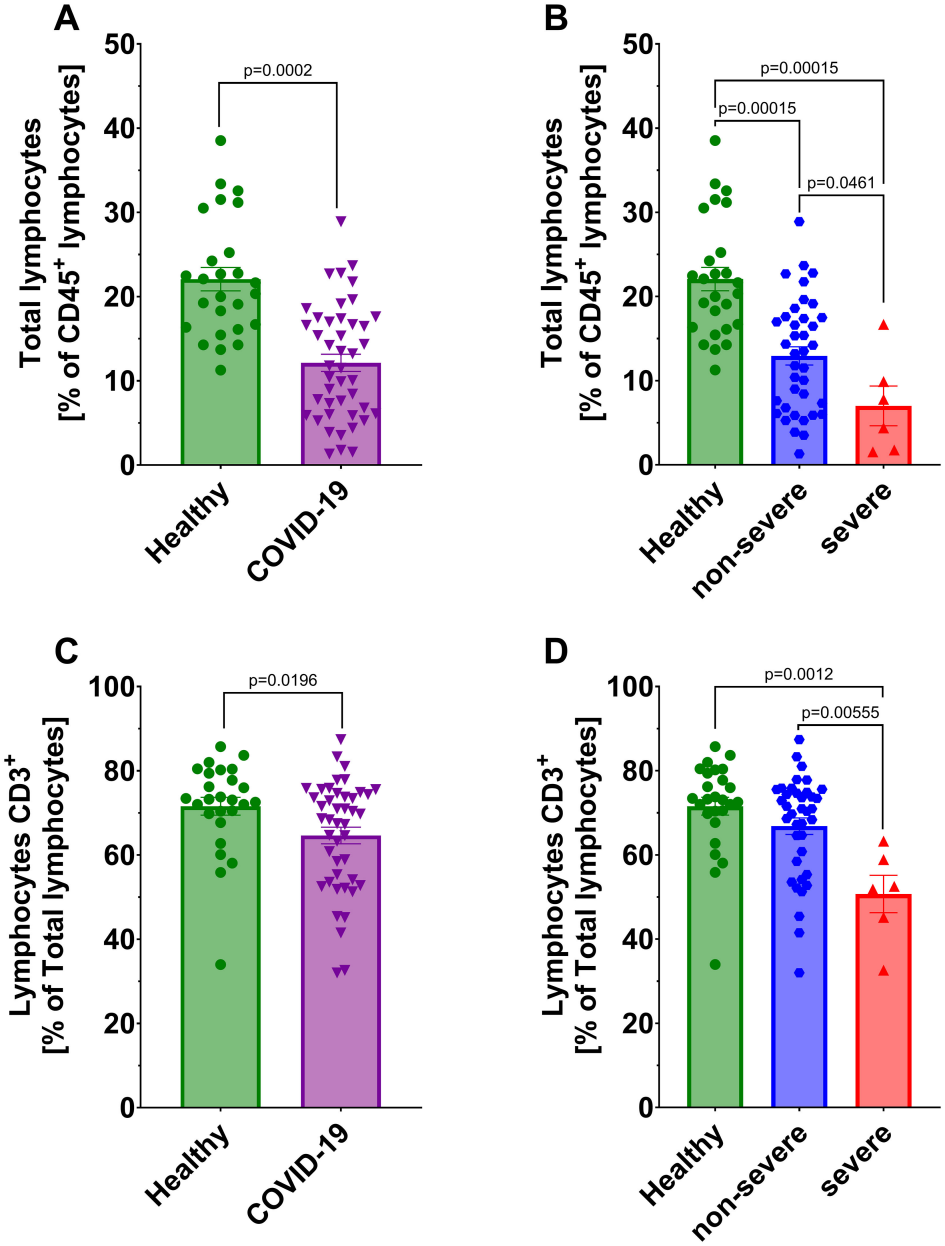
Analysis of total lymphocytes from CD45^+^ population between **(A)** healthy individuals (n=26) *and* all COVID-19 patients (n=44) and **(B)** healthy individuals (n=26) and COVID-19 patients divided into non-severe (n=38) and severe (n=6) COVID-19 groups according to the disease severity; Lymphocytes CD3^+^ from total lymphocytes population **(C)** healthy individuals (n=26) and all COVID-19 patients (n=44); **(D)** healthy individuals (n=26) and COVID-19 patients divided into non-severe (n=38) and severe (n=6) COVID-19 groups according to the disease severity. Lymphocyte population analysis was performed using flow cytometry. Each graph presents individual values along with the mean value ± SEM. Statistical significance levels are shown in the graphs.

**Figure 2:**
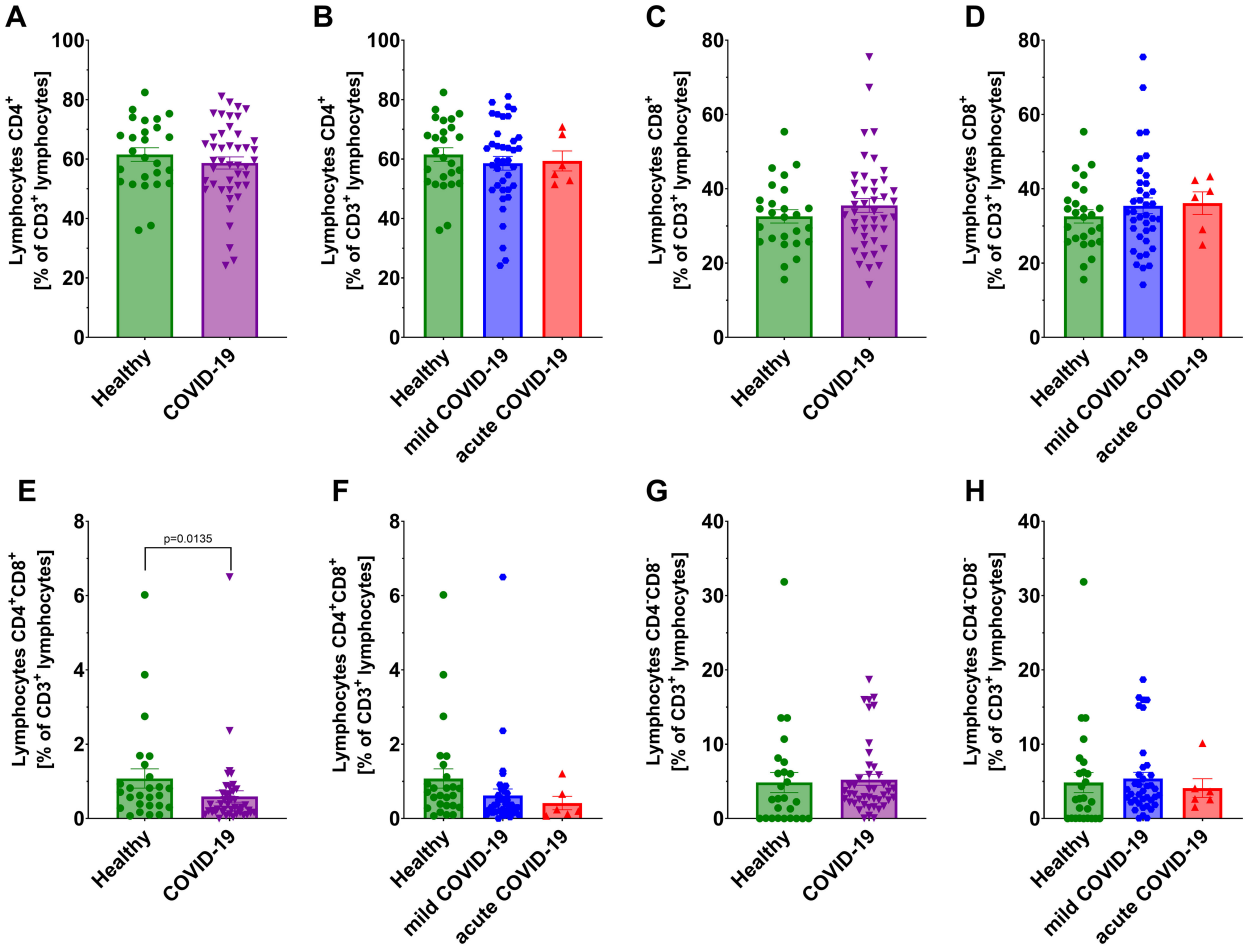
Analysis of T lymphocytes from CD3^+^ population: CD4^+^ lymphocytes in: **(A)** healthy individuals (n=26) *and* all COVID-19 patients (n=44); and **(B)** healthy individuals (n=26) and COVID-19 patients divided into non-severe (n=38) and severe (n=6) COVID-19 groups according to the disease severity; CD8^+^ lymphocytes **(C)** in healthy individuals (n=26) *vs* all COVID-19 patients (n=44); and **(D)** healthy individuals (n=26) and COVID-19 patients divided into non-severe (n=38) and severe (n=6) COVID-19 groups according to the disease severity; CD4^+^CD8^+^ lymphocytes **(E)** in healthy individuals (n=26) *vs* all COVID-19 patients (n=44) and **(F)** healthy individuals (n=26) and COVID-19 patients divided into non-severe (n=38) and severe (n=6) COVID-19 groups according to the disease severity; CD4^-^CD8^-^ lymphocytes in **(G)** healthy individuals (n=26) *and* all COVID-19 patients (n=44); and **(H)** healthy individuals (n=26) and COVID-19 patients divided into non-severe (n=38) and severe (n=6) COVID-19 groups according to the disease severity; CD8^+^ lymphocytes. Lymphocyte population analysis was performed using flow cytometry. Each graph presents individual values along with the mean value ± SEM. Statistical significance levels are shown in the graphs.

We observed significantly lower count of total lymphocytes and CD3^+^ T lymphocytes in all COVID-19 patients compared to healthy individuals (**Figures 1A, C**). A further significant decrease in these populations was also noted between non-severe and severe COVID-19 groups (**Figures 1B, D**). No significant differences were detected in CD4^+^ helper and CD8^+^ cytotoxic T lymphocytes, either between COVID-19 patients and healthy individuals or between non-severe and severe cases (**Figures 2A–D**). However, a significant reduction in double-positive lymphocytes (CD4^+^CD8^+^) was observed in all COVID-19 patients compared to healthy individuals (**Figure 2E**). No significant differences were detected when comparing the non-severe or severe groups with healthy individuals (**Figure 2F**). Similarly, no significant differences were observed for double-negative lymphocytes either (**Figures 2G, H**).

### 3.2 CD4^+^CD25^+/-^, CD4^+^CD45RA^+/-^ and CD8^+^CD45RA^+/-^ T lymphocyte subpopulations decrease in COVID-19 patients

**Figure 3:**
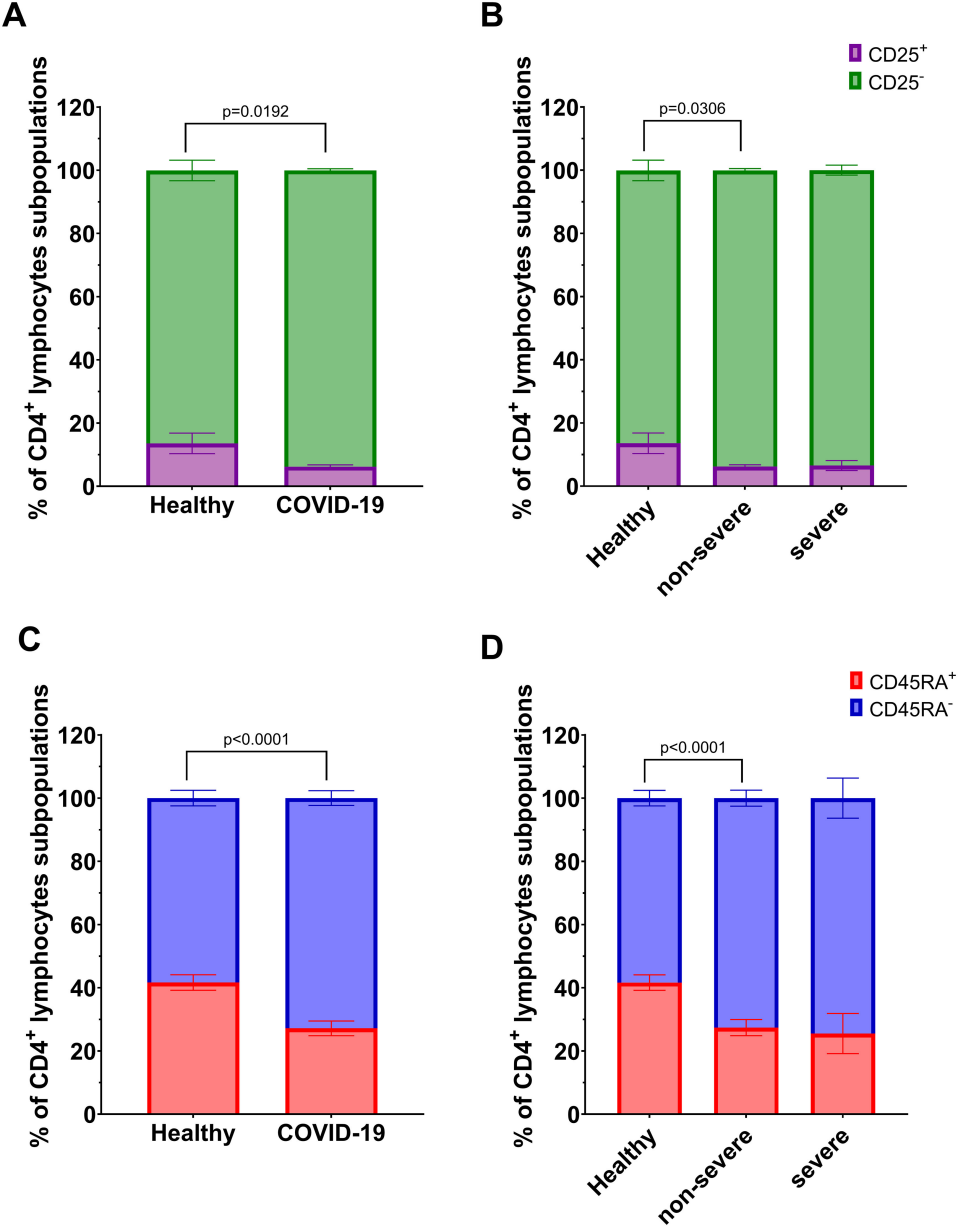
Analysis of subpopulations of CD4^+^ lymphocyte population: CD25^+^ *vs* CD25^-^ between **(A)** healthy individuals (n=26) and all COVID-19 patients (n=44); and **(B)** healthy individuals (n=26) and COVID-19 patients divided into non-severe (n=38) and severe (n=8) COVID-19 groups according to the disease severity; CD4^+^ lymphocytes; CD45RA^+^ *vs* CD45RA^-^ between **(C)** healthy individuals (n=26) and all COVID-19 patients (n=44); and **(D)** healthy individuals (n=26) and COVID-19 patients divided into non-severe (n=38) and severe (n=6) COVID-19 groups according to the disease severity. Lymphocyte population analysis was performed using flow cytometry. Each graph presents mean value ± SEM. Statistical significance levels are shown in the graphs.

We analyzed T lymphocyte subpopulations, defined as follows: CD4^+^CD25^+^, CD8^+^CD25^+^ (activated T lymphocytes), CD4^+^CD25^-^, CD8^+^CD25^-^ (effector T lymphocytes), CD4^+^CD45RA^+^, CD8^+^CD45RA^+^ (naïve T lymphocytes), and CD4^+^CD45RA^-^, CD8^+^CD45RA^-^ (memory T lymphocytes). Flow cytometry analysis showed a significant changes in the proportions of the population: decrease of CD4^+^CD25^+^ and CD4^+^CD45RA^+^ T lymphocytes, accompanied by an increase in CD4^+^CD25^-^ and CD4^+^CD45RA^-^ T lymphocytes in COVID-19 patients compared to healthy individuals (**Figures 3A, C**). These differences were also evident in non-severe group compared to the healthy individuals, but not in severe group (**Figures 3B, D**).

**Figure 4:**
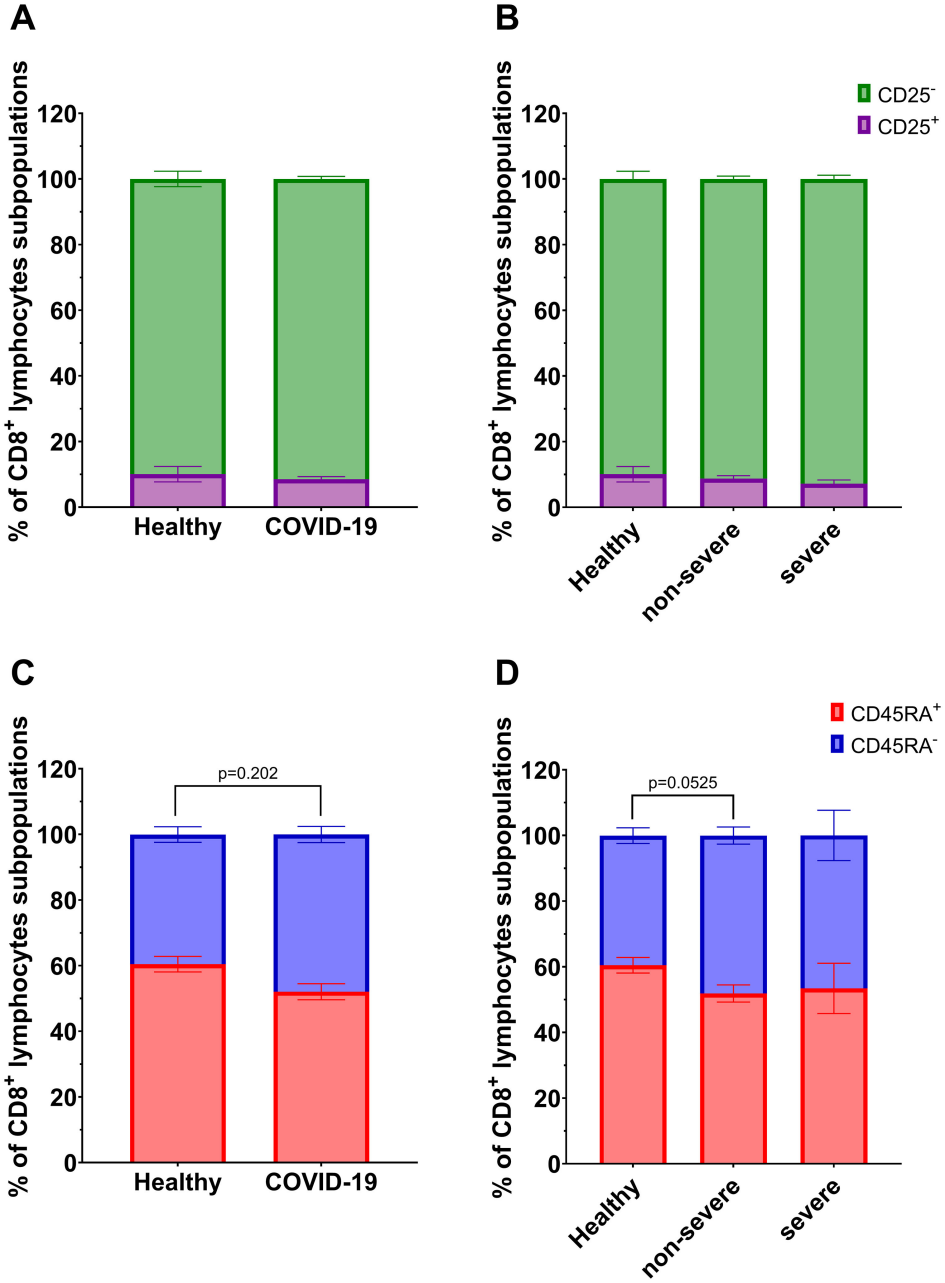
Analysis of subpopulations of CD8^+^ lymphocyte population: CD25^+^ vs CD25^-^ between **(A)** healthy individuals (n=26) and all COVID-19 patients (n=44); and **(B)** healthy individuals (n=26) and COVID-19 patients divided into non-severe (n=38) and severe (n=6) COVID-19 groups according to the disease severity; CD8^+^ lymphocytes; CD45RA^+^ vs CD45RA^-^ between **(C)** healthy individuals (n=26) and all COVID-19 patients (n=44); and **(D)** healthy individuals (n=26) and COVID-19 patients divided into non-severe (n=38) and severe (n=6) COVID-19 groups according to the disease severity. Lymphocyte population analysis was performed using flow cytometry. Each graph presents mean value ± SEM. Statistical significance levels are shown in the graphs.

Among CD8^+^ T lymphocyte subpopulations, a significant decrease in CD8^+^CD45RA^+^ lymphocytes and a corresponding increase in CD8^+^CD45RA^-^ lymphocytes were observed in COVID-19 patients compared to healthy individuals (**Figure 4C**). A similar tendency was also noted in the non-severe group compared to controls, although the difference did not reach statistical significance (p=0.0525, **Figure 4D**). No significant differences were observed for CD8^+^CD25^+^/CD25^-^ subsets (**Figures 4A, B**).

### 3.3 Regulatory T lymphocyte population decreases in COVID-19 patients

**Figure 5:**
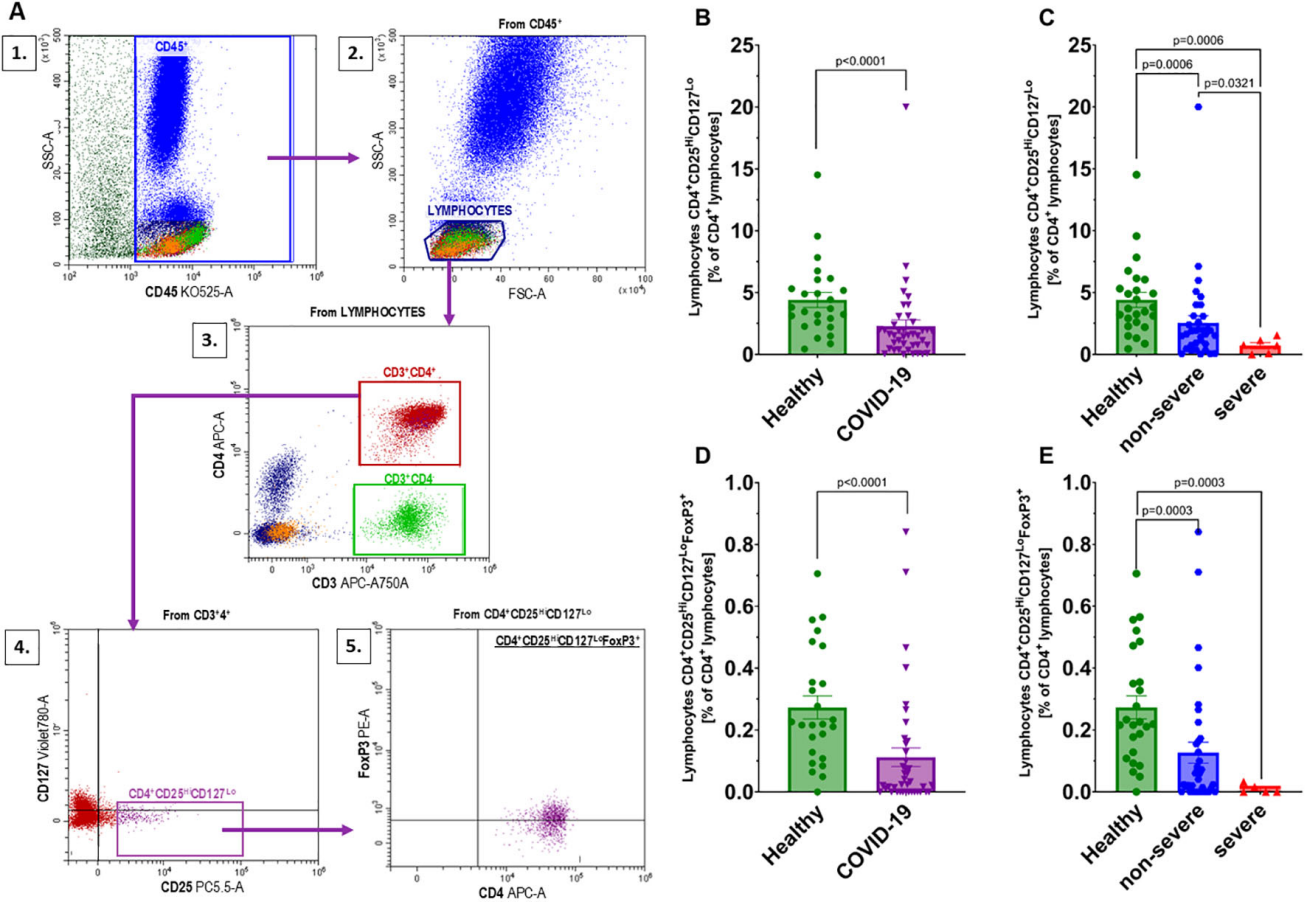
Analysis of regulatory T lymphocyte populations: **(A)** Gating strategy for regulatory T lymphocytes according to the following scheme: 1 – gating CD45^+^ cells from whole events; 2 – gating the population of lymphocytes from the CD45^+^ population; 3 – gating CD3^+^CD4^+^ from the lymphocyte population; 4 – gating CD4^+^CD25^Hi^CD127^Lo^ from the CD3^+^CD4^+^ population; 5 – gating FoxP3^+^ cells from the CD4^+^CD25^Hi^CD127^Lo^population; Changes in the population of CD4^+^CD25^Hi^CD127^Lo^cells between **(B)** healthy individuals (n=25) and all COVID-19 patients (n=41); and **(C)** healthy individuals (n=25) and COVID-19 patients categorized into non-severe (n=35) and severe (n=6) COVID-19 groups based on the disease severity; CD4^+^CD25^Hi^CD127^Lo^FoxP3^+^ designated from the population of CD4^+^CD25^Hi^CD127^Lo^lymphocytes in **(D)** healthy individuals (n=25) and all COVID-19 patients (n=40), and **(E)** healthy individuals (n=25) and COVID-19 patients categorized into non-severe (n=35) and severe (n=5) COVID-19 groups based on the disease severity. Lymphocyte population analysis was performed using flow cytometry. Each graph shows individual values along with the mean ± SEM. Statistical significance levels are shown in the graphs.

We investigated regulatory T lymphocytes (Treg), defined as CD4^+^CD25^Hi^CD127^Lo^ cells, and further identified a subset of natural regulatory T lymphocytes (nTreg) characterized as CD4^+^CD25^Hi^CD127^Lo^FoxP3^+^. The gating strategy used for identifying these populations in flow cytometry is shown in **Figure 5A**. A significant decrease was observed in the percentages of both CD4^+^CD25^Hi^CD127^Lo^ and CD4^+^CD25^Hi^CD127^Lo^FoxP3^+^ T lymphocytes in COVID-19 patients compared to healthy individuals (**Figures 5B, D**). When analyzed by disease severity, both non-severe and severe groups showed significantly reduced levels of these populations relative to healthy individuals (**Figures 5C, E**), with the lowest percentages found in patients with severe disease. However, due to the limited sample size, this difference should be interpreted cautiously.

### 3.4 Increased levels of IL6, IL10 and IFNγ in COVID-19 patients

**Figure 6:**
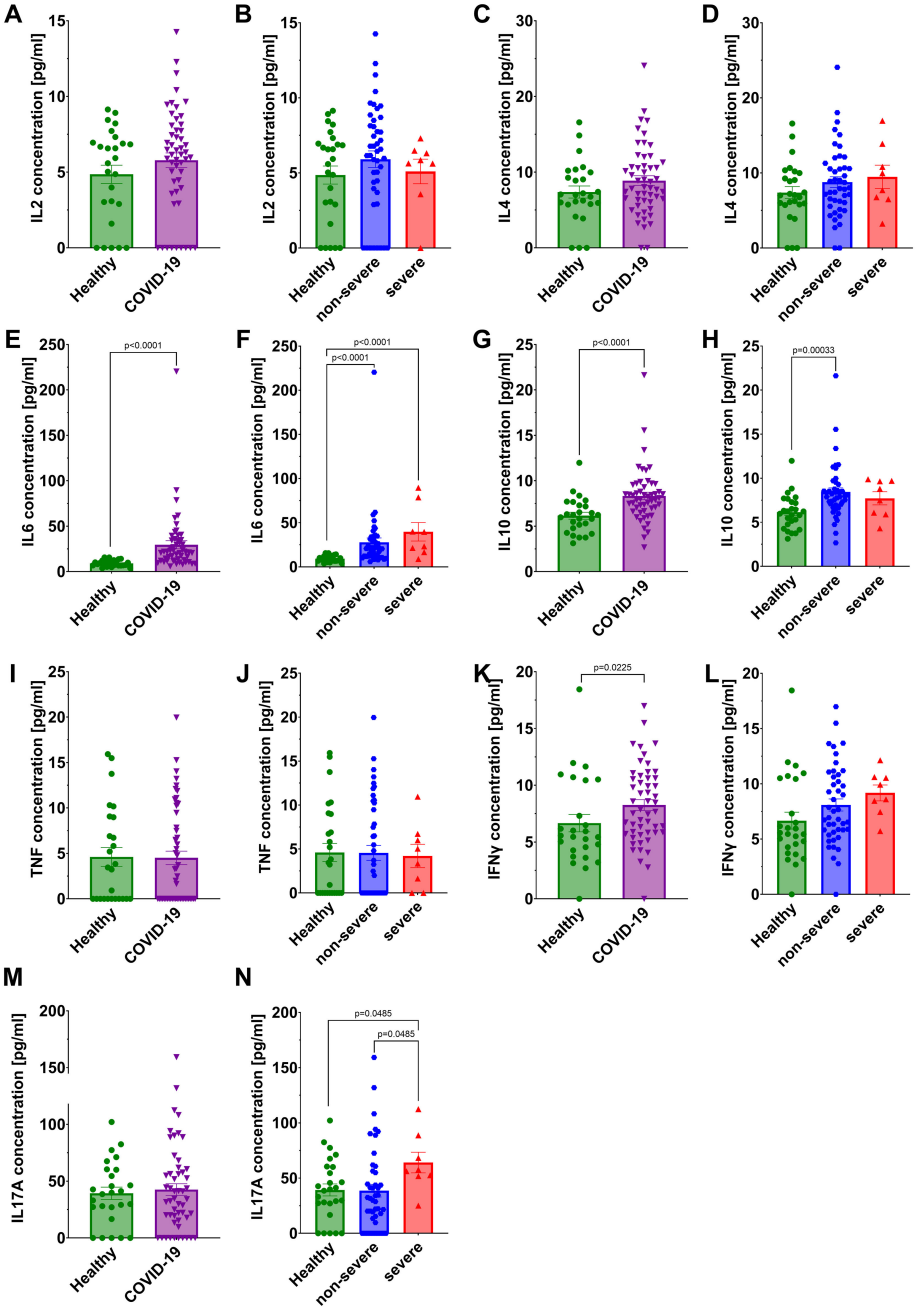
Analysis of cytokines concentration in the serum: IL2 in **(A)** healthy individuals *and* all COVID-19 patients and **(B)** healthy individuals and COVID-19 patients divided into non-severe and severe COVID-19 groups according to the disease severity; IL4 in **(C)** healthy individuals *and* all COVID-19 patients and **(D)** healthy individuals and COVID-19 patients divided into non-severe and severe COVID-19 groups according to the disease severity; IL6 in **(E)** healthy individuals *and* all COVID-19 patients and **(F)** healthy individuals and COVID-19 patients divided into non-severe and severe COVID-19 groups according to the disease severity; IL10 in **(G)** healthy individuals *and* all COVID-19 patients and **(H)** healthy individuals and COVID-19 patients divided into non-severe and severe COVID-19 groups according to the disease severity; TNF in **(I)** healthy individuals *and* all COVID-19 patients and **(J)** healthy individuals and COVID-19 patients diversified into non-severe and severe COVID-19 groups according to the disease severity; IFNγ in **(K)** healthy individuals *and* all COVID-19 patients and **(L)** healthy individuals and COVID-19 patients divided into non-severe and severe COVID-19 groups according to the disease severity; IL17A in **(M)** healthy individuals *and* all COVID-19 patients and **(N)** healthy individuals and COVID-19 patients divided into non-severe and severe COVID-19 groups according to the disease severity. Group sizes for all panels: healthy, n=26; non-severe COVID-19, n=44; severe COVID-19, n=8 (total COVID-19, n=52). Each graph shows individual values along with the mean value ± SEM. Statistical significance levels are shown in the graphs.

We observed a statistically significant increase in the concentrations of IL6, IL10, and IFNγ in the sera of all COVID-19 patients compared to healthy individuals (**Figures 6E, G, K**). For IL6 a significantly higher levels were found in both non-severe and severe groups relative to healthy individuals (**Figure 6F**). In the case of IL10, a significant increase was observed in the non-severe group compared to healthy individuals, whereas no difference was detected in the severe group (**Figure 6H**). IFNγ levels were elevated in the overall COVID-19 cohort compared to healthy individuals, but no significant differences were found after stratification by disease severity (**Figure 6L**). Interestingly, IL17A concentrations were significantly higher in the severe group compared to both healthy controls and non-severe patients (**Figure 6N**), but no significant differences were found beetween COVID-19 cohort in comparison to healthy individuals (**Figure 6M**). No statistically significant differences were detected in IL2, IL4, or TNF concentrations between healthy individuals and COVID-19 patients (**Figures 6A–D, I, J**). Given the limited number of patients in the severe group, these findings should be interpreted with caution.

### 3.5 Chemokine levels in COVID-19 patients

**Figure 7:**
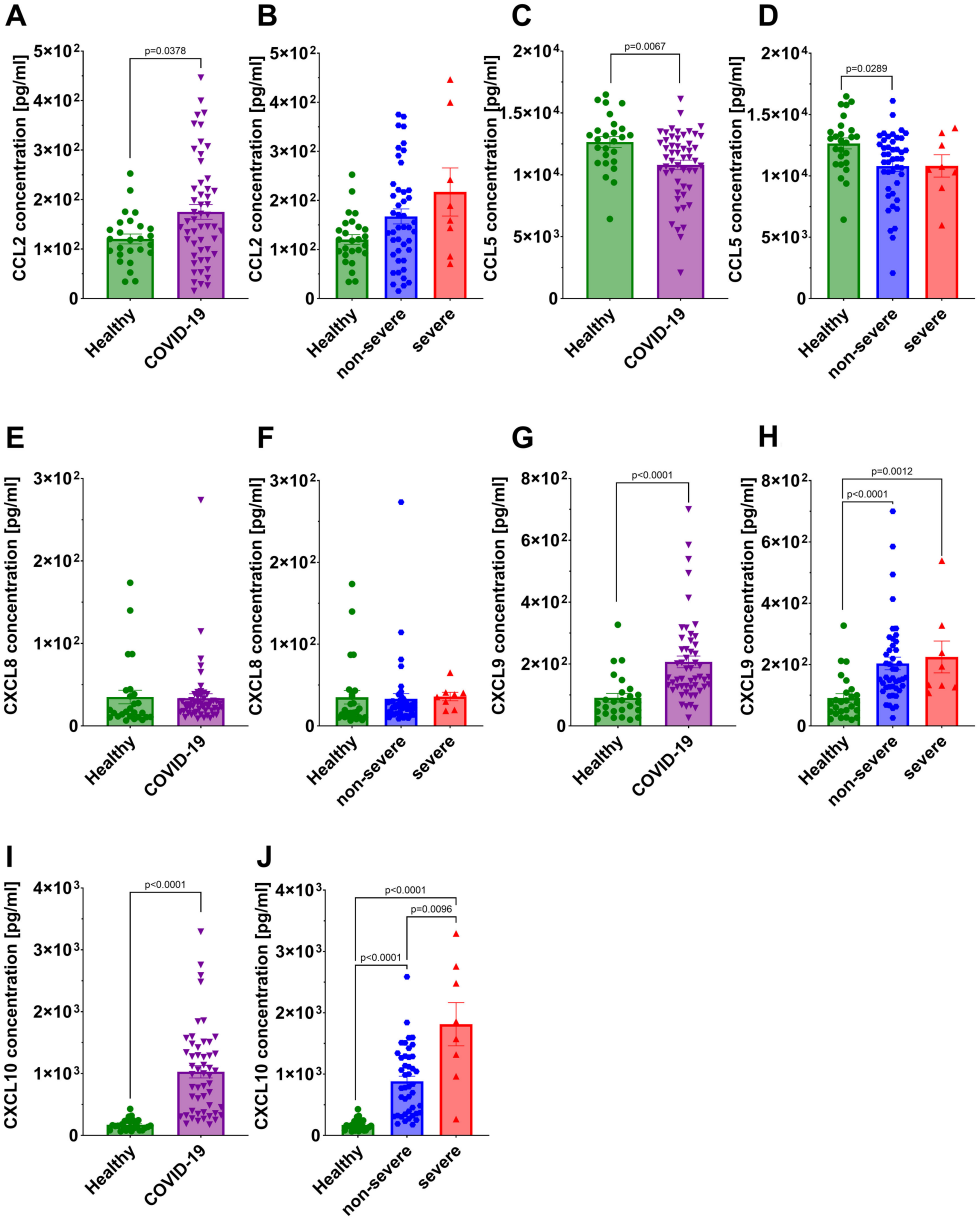
Analysis of chemokine concentration in the serum: CCL2 in **(A)** healthy individuals *and* all COVID-19 patients and **(B)** healthy individuals and COVID-19 patients divided into non-severe and severe COVID-19 groups according to the disease severity; CCL5 in **(C)** healthy individuals *and* all COVID-19 patients and **(D)** healthy individuals and COVID-19 patients divided into non-severe and severe COVID-19 groups according to the disease severity; CXCL8 in **(E)** healthy individuals *and* all COVID-19 patients and **(F)** healthy individuals and COVID-19 patients divided into non-severe and severe COVID-19 groups according to the disease severity; CXCL9 in **(G)** healthy individuals *and* all COVID-19 patients and **(H)** healthy individuals and COVID-19 patients diversified into non-severe and severe COVID-19 groups according to the disease severity; CXCL10 in **(I)** healthy individuals *and* all COVID-19 patients and **(J)** healthy individuals and COVID-19 patients divided into non-severe and severe COVID-19 groups according to the disease severity. Group sizes for all panels: healthy, n=26; non-severe COVID-19, n=44; severe COVID-19, n=8 (total COVID-19, n=52). Each graph shows individual values along with the mean value ± SEM. Statistical significance levels are shown in the graphs.

A statistically significant reduction in CCL5 concentration was noted in all COVID-19 patients compared to the healthy individuals (**Figure 7C**), with the decrease being significant only in the non-severe group after stratification (**Figure 7D**). In contrast, a statistically significant increase in concentration of CCL2, CXCL9 and CXCL10 was observed in all COVID-19 patients compared to the healthy controls (**Figures 7A, G, I**). After stratifying patients by disease severity, significant increases were observed for CXCL9 (both non-severe and severe groups vs healthy) and CXCL10 (non-severe and severe groups vs healthy), whereas CCL2 did not differ significantly in either subgroup (**Figures 7B, H, J**). CXCL8 concentrations did not differ significantly between healthy controls and either COVID-19 subgroup (**Figures 7E, F**). Interestingly, CXCL10 concentrations were significantly higher in severe COVID-19 patients compared to the non-severe group (**Figure 7J**). Among the analyzed chemokines, CXCL10 was the only one that differentiated between non-severe and severe COVID-19, with mean serum concentrations of 205 pg/ml in healthy individuals, 883 pg/ml in the non-severe group, and 1813 pg/ml in the severe group. Given the limited size of the severe cohort, these findings should be interpreted with caution.

### 3.6 Correlation matrix analysis

**Figure 8:**
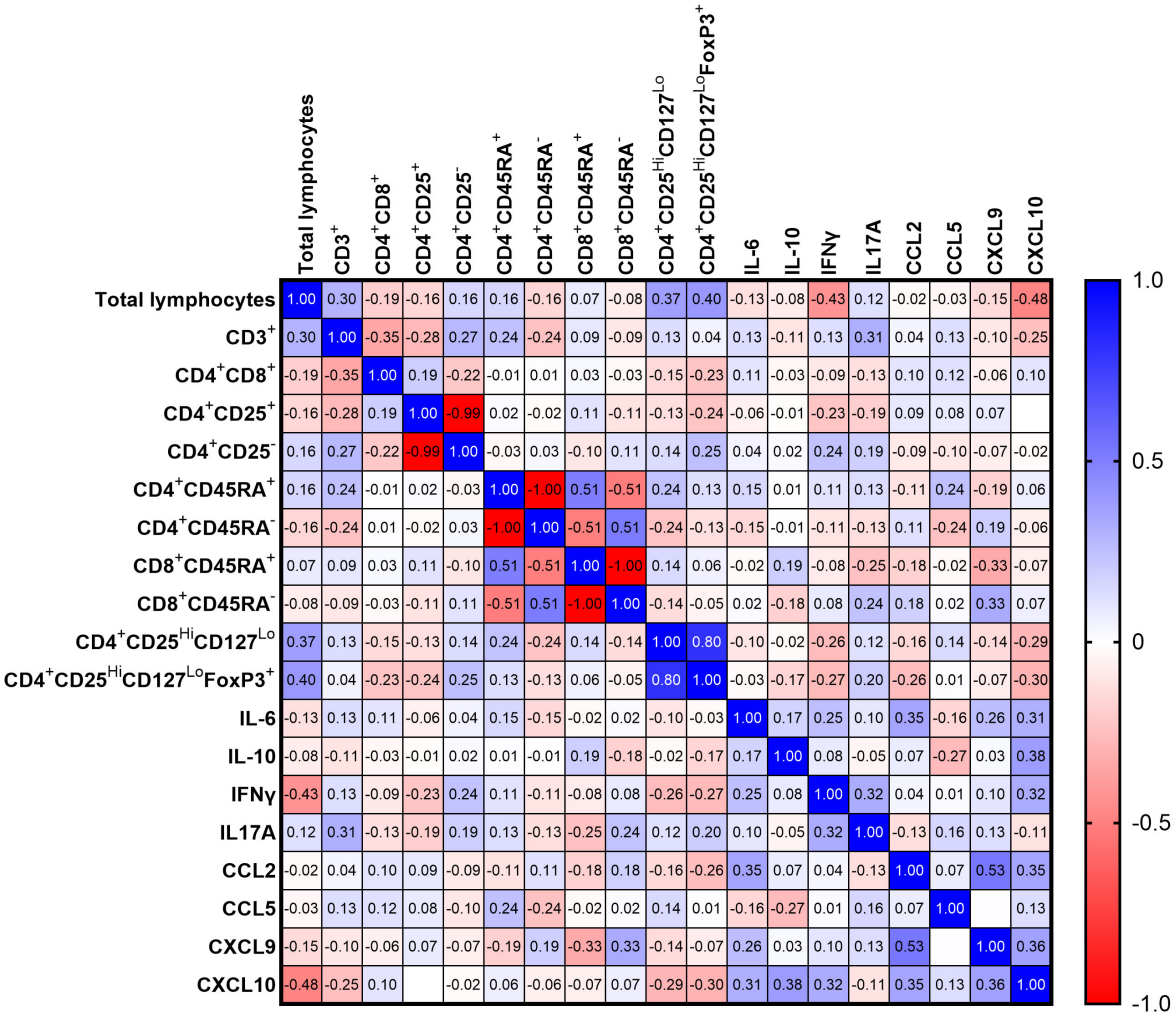
Heatmap representing Spearman R correlation for significantly changing variables for all COVID-19 patients (n=52); red tones - positive correlation; blue tones - negative correlation; strong correlation - values close to 1 or -1; no correlation - values equal to 0.

The correlation matrix presented in **Figure 8** illustrates the relationships between 19 statistically significant variables analyzed in this study among the 52 COVID-19 patients. Spearman’s correlation coefficient (r) was calculated for each pair of variables. The chemokine CXCL10 showed the highest number of statistically significant correlations among all variables included in the analysis. We observed a negative correlation between CXCL10 and total lymphocytes count (*r=-0.48*; *p=0.0009*) and positive correlation with IL6 (*r=0.31; p=0.025*), IFNγ (*r=0.32*; *p=0.02*), CCL2 (*r=0.35*; *p=0.01*) and CXCL9 (*r=0.36; p≤ 0.008*). A trend toward significance was also observed for CXCL10 and IL10 (r = 0.38; p = 0.053). Additionally, we found positive correlations between total lymphocytes and CD4^+^CD25^Hi^CD127^Lo^ (*r=0.37*; *p* = 0.017) as well as CD4^+^CD25^Hi^CD127^Lo^FoxP3^+^ (*r=0.40*, *p ≤* 0.011). A negative correlation was observed between total lymphocytes and IFNγ (*r=-0,43; p=0.003*). We also observed significant correlations for CD3^+^ with IL17A (*r=-0,31; p=0.04*), CXCL9 with CD8^+^CD45RA^+^ lymphocytes (*r=-0,33; p=0,03*), CCL2 with IL6 (*r=0.35; p=0.01*) and CXCL9 with CCL2 (*r=0,53; p≤ 0.001*).

## 4 Discussion

WHO’s declaration states that SARS-CoV-2 outbreaks continue to occur globally, still causing life-threatening conditions—particularly in unvaccinated individuals. In 2024, new outbreaks were reported, though they were not widespread across multiple continents (15–17). On March 28, 2025, the European Centre for Disease Prevention and Control updated its list of SARS-CoV-2 variants of concern, including Omicron, KP.3 and BA.2.86, while Omicron XEC and LP.8.1 remain under consistent monitoring (18). Since the emergence of SARS-CoV-2, numerous studies have investigated immune response parameters, particularly immune cells, cytokines and chemokines, in relation to the clinical progression of COVID-19 (19–23).

In our study, we analyzed the immunological profiles of hospitalized COVID-19 patients during the 2021 pandemic in Warsaw, Poland, to identify potential biomarkers associated with immune dysregulation and prospective disease severity. Such biomarkers should be an early prognostic indicators of COVID-19 progression. Identifying them is important for predicting disease course and enabling timely, individualized therapeutic strategies.

We screened patients with severe and non-severe COVID-19 for selected subsets of T lymphocytes, cytokines specific to Th1/Th2/Th17 responses, and chemokines from the CC and CXC families, and compared the results with those of healthy volunteers. Samples were collected within 24 h of hospital admission, prior to the onset of symptoms used for stratifying patients into non-severe or severe groups.

Changes in lymphocytes percentages in the blood are often among the first signs of inflammation, with disease progression reflected by shifts in the distribution of various lymphocyte populations. In our study, we observed that the population of total lymphocytes and CD3^+^ T lymphocytes decreased in a severity-dependent manner, consistent with previously reported findings (24–28). However, we did not observe any significant changes in the percentage of helper T cells (CD4^+^) or cytotoxic T cells (CD8^+^), in contrast to other studies (24, 25, 27, 29). These discrepancies are unlikely to be explained by methodological differences, as standard and widely accepted techniques were used. Instead, population-specific factors and/or the influence of group size in the non-severe/severe cohorts may have contributed to the observed differences.

Recent studies have shown that double-positive lymphocytes may be involved in the pathogenesis of HIV (30), Dengue virus (31), Hantaan virus (32) and Hepatitis C virus (33). In our study, the double-positive lymphocytes population was significantly decreased in all COVID-19 patients compared to healthy individuals. These findings are consistent with the study by Kalpakci et al. (24) but not with Zahran et al. results (26). The role of double-positive lymphocytes in SARS-CoV-2 infection therefore remains unclear and requires further investigation. Moreover, double-positive lymphocytes are not considered as suitable biomarkers for COVID-19 severity, as their population is relatively small, and consequently difficult to identify.

Naïve T lymphocytes, precursors to memory and effector T cells, circulate in the periphery until activated by new antigens-presenting cells (APCs) (34). In our study, we observed a modest yet statistically significant decrease in the naïve CD4^+^CD45RA^+^/CD8^+^CD45RA^+^ T lymphocyte population in COVID-19 patients. Previous studies have reported an increase in the naïve CD4^+^CD45RA^+^ subpopulation in patients with non-severe and severe COVID-19, which contrasts with our findings (35–37). Since naïve T lymphocytes are crucial for maintaining a diverse T cell receptor (TCR) repertoire to recognize a broad range of pathogens (36), even a slight reduction in COVID-19 cases could suggest early signs of impaired immune responsiveness. However, given the relatively small effect size, this finding should be interpreted cautiously and warrants confirmation in larger cohorts.

Treg lymphocytes play an important role in modulating the immune response. Their increased activity can attenuate immune effectiveness, while they also prevent excessive immune activation by limiting effector lymphocyte activation and regulating cytokine production (38, 39). Patients in both the non-severe and severe groups showed a reduced percentage of Treg lymphocytes CD4^+^CD25^Hi^CD127^Lo^ and nTreg lymphocytes with FoxP3 expression CD4^+^CD25^Hi^CD127^Lo^FoxP3^+^, which are widely recognized as subpopulations with regulatory functions (40). The available literature presents a heterogeneous depiction of the dynamic changes in Treg populations, as COVID-19 patients have been described with increased (41–44), decreased (23, 25, 36) or no change (27, 45–47) in this lymphocyte populations. Similarly to our results, Jiménez-Cortegana et al. (29) observed a reduced percentage of Treg lymphocytes with the CD4^+^CD25^Hi^CD127^Lo^ phenotype in COVID-19 patients compared to healthy controls. In contrast, Dai et al. (28) and Fentoligo et al. (27) reported no differences in percentage of the Treg lymphocyte with CD4^+^CD25^+^FoxP3^+^ phenotype between patients with different COVID-19 severity (28) and between patients with severe COVID-19 compared to healthy individuals (27). Additionally, Wang et al. (48) showed that, the percentage of Treg lymphocyte with a CD4^+^CD25^+^CD127^-^ phenotype increased during progression from mild to severe conditions, then decreased during progression from severe to critical conditions. These discrepancies may result from the different criteria used for identifying Treg lymphocytes and the varying sampling timelines during the infection course. Moreover, differences may also arise from the use of whole blood cells (25, 28, 49)) versus peripheral blood mononuclear cells (23, 27, 47). Our findings suggest that impaired Treg lymphocyte function may contribute to disease progression and severity, but this requires confirmation in larger cohorts.

Hyperactivation of T lymphocytes and the excessive release of pro-inflammatory cytokines increase vascular permeability and plasma leakage, ultimately leading to lung injury, severe respiratory distress syndrome, and multi-organ failure (50–53). This process is recognized as a major contributor to severe COVID-19 and the associated mortality. In our study, we demonstrated that SARS-CoV-2 infection caused no significant differences in concentrations of IL2, IL4, TNF and CXCL8 in the sera of COVID-19 patients compared to healthy volunteers. However, a significant increase was observed in the anti-inflammatory IL10, as well as the pro-inflammatory cytokines IL6, IFNγ and IL17A, and the pro-inflammatory chemokines CCL2, CCL5, CXCL9, CXCL10 in patients with both non-severe and severe COVID-19. Notably, CXCL10 exhibited a severity-dependent pattern, progressively increasing over the course of the disease. Overall, the observed cytokine and chemokine increases appeared to be associated with disease severity, although the relatively small cohort size call for cautious interpretation and confirmation in larger studies.

The available literature extensively describes changes in cytokine and chemokine concentrations in serum (19, 54, 55) and plasma (21, 56, 57) in patients with varying severity of COVID-19. Bourhis et al. (55) reported increased serum concentrations of IL4, IL6, IL10 in SARS-CoV-2 infected patients compared to healthy controls, with the latter cytokines being higher in severe and critical cases. They also showed increased concentrations of the CCL2 and CXCL10 in accordance with disease progression, consistent with our data (55). Similarly, Ravindran et al. (21) demonstrated that plasma concentrations of IL6 and CXCL10 were significantly elevated in patients with severe COVID-19. Huang et al. (20), identified IL6, IL10 and CXCL10 as factors strongly associated with progression from mild to severe disease. Trifonova et al. (19), reported that CXCL8 and CXCL10 distinguished mild from moderate/severe cases, although not at statistically significant levels, while IL6 and IL10 concentrations were significantly higher in severe compared to mild and moderate cases. Zuñiga et al. (58), described dynamic changes in CXCL10 concentrations in the sera of Zika virus patients: levels were significantly elevated from enrollment (day 0) until day 28 compared to healthy controls, peaking on days 0 and 3, and decreasing by days 7 and 28. CXCL10 also appears to be an important factor in other viral diseases, such as SARS and measles, where the concentration of this chemokine is elevated and increases with disease progression, while a decreases are associated with recovery (59–61). Taken together, these observations suggest that CXCL10 may be a broadly relevant marker of immune activation in viral infections. However, its potential role as a diagnostic or prognostic biomarker in COVID-19 requires further clarification through studies using sequentially collected samples and larger, representative patient cohorts.

CXCL10 is a chemokine secreted by various cell types e.g.: fibroblasts, endothelial cells, T lymphocytes and monocytes/macrophages. It binds to the CXCR3 receptor, expressed on immune cells such as T and B lymphocytes, and its activation promotes chemotaxis, proliferation, and recruitment of macrophages, Th1 lymphocytes, and NK cells (22). This increased leukocyte activity may contribute to systemic inflammation and tissue damage (22). In our study, CXCL10 differentiated patients with a severe disease course from those with non-severe disease and from healthy controls. CXCL10 levels significantly correlated with total lymphocyte count and with IL6, IFNγ, CCL2, and CXCL9. Rydyznski Moderbacher et al. (62) also showed that CXCL10 strongly correlated with disease severity and the number of CD4^+^ and CD8^+^ T cells, while Lore et al. (63) demonstrated positive correlation between CXCL10 and CCL2, IFNγ, IL1Ra, CCL5, CCL11, IL6. Additionally, Fabris et al. (64) reported additive correlations between IL6 and IL10, IL10 and CXCL10, TNFα and CXCL8.

In our cohort, CXCL10 showed consistent associations with disease severity and with several other immune parameters, suggesting its involvement in the broader dysregulation of the immune response in COVID-19. While these findings highlight CXCL10 as a potentially informative biomarker, its role should be interpreted with caution given the limited sample size and the complexity of the cytokine network. Validation in larger and more diverse patient populations will be necessary to establish whether CXCL10, alone or as part of a biomarker panel, can reliably predict COVID-19 severity.

In our study CXCL10 was identified as a crucial chemokine in the severe course of COVID-19, serving as an early marker of diseases progression. Additionally, the correlation between CXCL10 and the percentage of total lymphocytes, as well as the levels of IL6, IFNγ, CCL2 and CXCL9 suggests a complex immune dysregulation occurring in COVID-19. This may serve as an associated panel for predicting the severity of disease.

Our single-center study was conducted during the COVID-19 pandemic in 2021 among hospitalized, unvaccinated patients. At that time in Poland, infections were mainly caused by the Alpha, Beta, and Delta variants, and successive age and occupational groups of the population were gradually included in vaccination programs. Since the study was conducted, the immunological landscape has continued to evolve with the emergence of new SARS-CoV-2 variants, such as Omicron, as well as due to widespread vaccination. The predictive value of biomarkers identified on the basis of 2021 data may not be the same for infections caused by currently circulating SARS-CoV-2 variants and in vaccinated individuals. However, some of the biomarkers we identified may remain relevant regardless of vaccination status and viral variant. Kawasuji et al. (65) demonstrated no statistically significant differences in serum levels of CXCL10, IL6, and IFNγ when comparing vaccinated and unvaccinated patients. Moreover, other studies indicate that some of the biomarkers we analyzed also show significantly higher levels in patients with severe SARS (CXCL10, CCL2, IFNγ) and MERS (CXCL10, CCL2, IL6) compared to patients with mild forms of these diseases (66–70). These observations may point to the stability of certain biomarkers irrespective of vaccination and SARS-CoV-2 variants. Furthermore, the repeated involvement of CXCL10 and CCL2 in severe SARS, MERS, and COVID-19 may indicate shared mechanisms of inflammatory responses in severe coronavirus infections. In summary, further studies in representative patient cohorts are needed to confirm whether the biomarkers identified in the present work remain useful for risk stratification in today’s predominantly vaccinated populations and with respect to currently circulating variants. The identification of universal biomarkers could facilitate the assessment of the risk of severe COVID-19 and the implementation of potential interventions aimed at restoring immunological balance in affected patients.

## Data Availability

The raw data supporting the conclusions of this article will be made available by the authors, without undue reservation.
